# Development and Clinical Evaluation of an mHealth Application for Stress Management

**DOI:** 10.3389/fpsyt.2016.00130

**Published:** 2016-07-26

**Authors:** Brent D. Winslow, George L. Chadderdon, Sara J. Dechmerowski, David L. Jones, Solomon Kalkstein, Jennifer L. Greene, Philip Gehrman

**Affiliations:** ^1^Design Interactive, Inc., Orlando, FL, USA; ^2^Quantified Design Solutions LLC, Orlando, FL, USA; ^3^Philadelphia VA Medical Center, University of Pennsylvania, Philadelphia, PA, USA

**Keywords:** stress, electrodermal response, heart rate, mobile applications, wearable devices, cognitive behavioral therapy, telemedicine

## Abstract

A large number of individuals experience mental health disorders, with cognitive behavioral therapy (CBT) emerging as a standard practice for reduction in psychiatric symptoms, including stress, anger, anxiety, and depression. However, CBT is associated with significant patient dropout and lacks the means to provide objective data regarding a patient’s experience and symptoms between sessions. Emerging wearables and mobile health (mHealth) applications represent an approach that may provide objective data to the patient and provider between CBT sessions. Here, we describe the development of a classifier of real-time physiological stress in a healthy population (*n* = 35) and apply it in a controlled clinical evaluation for armed forces veterans undergoing CBT for stress and anger management (*n* = 16). Using cardiovascular and electrodermal inputs from a wearable device, the classifier was able to detect physiological stress in a non-clinical sample with accuracy greater than 90%. In a small clinical sample, patients who used the classifier and an associated mHealth application were less likely to discontinue therapy (*p* = 0.016, *d* = 1.34) and significantly improved on measures of stress (*p* = 0.032, *d* = 1.61), anxiety (*p* = 0.050, *d* = 1.26), and anger (*p* = 0.046, *d* = 1.41) compared to controls undergoing CBT alone. Given the large number of individuals that experience mental health disorders and the unmet need for treatment, especially in developing nations, such mHealth approaches have the potential to provide or augment treatment at low cost in the absence of in-person care.

## Introduction

Recent estimates suggest that approximately one-third of individuals globally will experience mental health disorders in their lifetime (1). Individuals in developing nations are particularly vulnerable (2, 3). In developed nations, an especially vulnerable population is military veterans. For example, approximately one-third of US military veterans suffer from some type of psychological distress, including post-traumatic stress disorder (PTSD), major depressive disorder (MDD), and suicidal ideation (4). Diagnostic and subthreshold levels of PTSD are associated with poor quality of life, including anger, stress, alcoholism, depression, poor physical health, and increased suicidality (5, 6), and cause impaired ability to function in social, educational, and work environments (7).

Among various interventions to treat depression and anxiety, cognitive behavioral therapy (CBT) has emerged as standard practice for reduction of psychiatric symptoms (8), with previous studies indicating that CBT has similar therapeutic effects as anti-depressant medication (9). CBT is generally administered by mental health professionals and consists of a structured, collaborative process that helps individuals consider and alter their thought processes and behaviors associated with stress or anxiety, usually administered weekly over several months (10). However, standard CBT for stress and anxiety does not offer the provider information regarding therapeutic efficacy outside of office visits nor does it provide objective information about individuals’ triggers, such as location, time, or severity (11). In addition, high dropout rates from CBT programs have been reported to span from 25% (12) to as high as 40% (13) for individuals suffering from depression. The limitations of CBT, including lack of objective data available for providers and high patient dropout rates, could be mitigated with emerging technologies. To support real-time objective stress monitoring in mental health treatment, wearable physiological sensors and associated mobile health (mHealth) applications (14) have the potential to quantify biological metrics associated with stress (15), support remote monitoring, and alert the wearer or provider to real-time changes in emotional state.

Existing approaches to stress detection use a wide array of features calculated from sensor data measuring various aspects of heartbeat, including pulse photoplethysmography (PPG) or ECG (15–17), skin conductance measurement (18–20), and measurement of respiration, all of which are responsive to increased sympathetic nervous system activity associated with stress (21). Standard supervised machine learning methods have been used previously to develop stress classifiers, which require subjects to engage in tasks known to induce stress so that stress or non-stress labels can be assigned to the input features. Previous work has emphasized the difficulties imposed on stress classification by individual subject variability in physiological responses to stress (16, 19). Another concern is the physical activity of subjects that triggers similar cardiovascular and electrodermal physiological signals as stress, leading to masking and confounds of stress detection (15, 19). The major challenge in using mobile physiological sensors to quantify stress is the lack of robust and clinically tested algorithms to classify stress in a mobile environment in real time (22).

Previous stress monitoring algorithms have been built with traditional laboratory physiological sensor suites that do not translate well to operational settings (17, 19), such as mental health treatment. New wearable devices with clinical grade sensors and associated mobile applications have the potential to take real-time stress monitoring outside of the laboratory. There is an opportunity to combine foundational mobile stress monitoring algorithm research methods with new mobile physiological sensor suites to create an accurate, quantitative classifier for continuous and objective real-time stress assessment. In the current study, we develop a physiological classifier of stress and apply it in a clinical evaluation of patients undergoing CBT for stress/anger management, following military deployment. It is hypothesized that stress induced using standard methods can be classified with high accuracy using a machine learning algorithm and that the use of such an algorithm in an mHealth application can reduce post-deployment psychiatric symptoms, including stress, anger, and anxiety, in a clinical population undergoing CBT.

## Materials and Methods

### Classifier Study

#### Participants

All methods involving participants were approved by a series of Institutional Review Boards [Copernicus Group IRB, Durham, NC, USA; Human Studies Subcommittee, Department of Veteran’s Affairs, Philadelphia, PA, USA; US Army Medical Research and Material Command Human Research Protection Office (HRPO), Fort Detrick, MD, USA].

Thirty-five participants (24 males; average age 25.7 ± 6.2 years) were recruited for the initial classifier-development study, which lasted approximately 1.5 h. Participants were recruited using recruitment flyers posted online and through recruitment fairs at local universities.

#### Experimental Procedure

Upon arrival, participants provided written-informed consent and completed a series of questionnaires including: demographics; the subjective units of distress scale (SUDS); the depression, anxiety, and stress scale (DASS); and the patient-reported outcomes measurement information scale (PROMIS) anger scale. Wireless physiological sensors were then placed on the participants, followed by a 5-min recording of baseline physiological activity, while participants remained seated. Participants then completed the Trier Social Stress Test [TSST; Ref. (23)]. The TSST was used to elicit physiological stress, consisting of 5 min each of: preparatory anticipatory stress (TSST-P); oral speech (TSST-S); and mental arithmetic (TSST-A). Following data acquisition, participants were debriefed and thanked for their participation. A subset of participants (*n* = 7) also provided a saliva sample *via* passive drool for cortisol assessment at baseline and following the TSST.

#### Qualitative Measures

Participants in the classifier study responded to the SUDS, in which they reported their current level of stress on a scale of 0–100, with 0 indicating that they were completely relaxed and 100 indicating that they were experiencing severe stress (24). Participants then completed the DASS, designed to assess current depression, anxiety, and stress using responses to 21 statements (25). Respondents indicated the degree to which each statement has been true for them over the preceding week on a 4-point Likert scale. Participants also responded to the PROMIS anger scale, which consists of an 8-item measure on which respondents indicate the frequency of each item from the past week on a 5-point Likert scale (26).

#### Physiological Measures

The Biopac MP-150 system (Goleta, CA, USA) was used for collection of physiological data. Participants were fitted with PPG at the non-dominant thumb and electrodermal activity (EDA) on the fourth and fifth fingers of the non-dominant hand, with band limits set between DC and 10 Hz. All physiological data were sampled at 1000 Hz and wirelessly sent to an MP-150 system running AcqKnowledge software (Biopac Systems, Goleta, CA, USA). Following data collection, PPG data were downsampled to 64 Hz and EDA was downsampled to 4 Hz. Heart rate (HR) was calculated from the R–R interval from the PPG signal, with intervals <40 and >180 bpm excluded from the analysis. Salivary cortisol was measured by standard ELISA (Salimetrics, Carlsbad, CA, USA; intra-assay CV = 4.5%, inter-assay CV = 5.8%).

In a subset of participants (*n* = 8), the Empatica E3 sensor was also used for physiological data collection. A second system was used to ensure the stress algorithm was compatible across multiple hardware solutions and to provide for mobile stress classification in future studies. Physiological data, consisting of PPG (64 Hz) and EDA (4 Hz), were transmitted *via* Bluetooth 4.0 to a custom mobile application for data collection implemented in the Android OS on a Samsung Galaxy S4 phone.

#### Classifier Development

Event times and physiological data were stored in Biopac.acq files. All data were read into Python analysis scripts running under the Enthought Canopy environment. The numpy, scipy, pandas, and matplotlib libraries were used for feature extraction and data analysis (27), and the scikit-learn library (28, 29) was used for classifier development. Visual inspection of the raw data in the Biopac software and the interactive Python environment was used to discard physiologically noisy or missing participant data.

From the raw data, non-overlapping 1-min windows were analyzed to yield feature vectors for the minute blocks. Inter-beat intervals (IBI) were extracted from the PPG data using a signal derivative-based algorithm (30). For minutes with less than 40 valid IBI samples, the block of data was discarded; for remaining blocks, the mean IBI was calculated. For each valid IBI block, the mean HR was estimated by dividing 60 by the IBI mean. For the EDA data, the mean was taken over the minute’s raw data. The HR and EDA means were then normalized separately for each participant by subtracting the average of the 5-min baseline.

Matplotlib boxplots and scatterplots were used to explore the distributions of the task-specific patterns (e.g., baseline and TSST-S) in feature space. A stress vs. non-stress classifier was trained using baseline vs. TSST-S, using baseline-normalized mean HR and EDA features. A 2-feature linear model classifier was trained and tested on the E3 dataset using stochastic gradient descent. The train:test (75:25%) set consisted of data feature vectors taken from the baseline and TSST-S minutes of the participants with E3 data. Five-fold cross-validation was implemented to evaluate the average performance of the algorithm on the train set: this set was divided into fifths and, iteratively, one of the five blocks was left out for testing and the other four were used to train the classifier. While the cross-validation measures were used to compare performance of different learning algorithms (e.g., stochastic gradient descent vs. support vector machines), the training and testing accuracies were calculated from performance from training on the full train set. Performance of the E3 data-trained model was also measured using the entire Biopac data set with good HR and EDA baseline and TSST-S minute data as a test set.

#### Data Analysis and Statistics

Classifier training accuracy was defined by signal detection theory (31) (hit = classifier correctly identified spike stress state; miss = classifier missed a spike in stress state; false alarm = classifier identified a spike in stress state when one did not actually occur; and correct rejection = classifier did not identify a spike in stress state when one did not occur). Accuracy was defined as the ratio of the sum of hits and correct rejections over the sum of minute blocks classified as either stressed or non-stressed. The hit rate was defined as the number of hit minute blocks divided by the total number true stress blocks (TSST-S), and the false-alarm rate was defined as the number of false-alarm blocks divided by the total number of true non-stress blocks (baseline). SUDS scores between baseline and the TSST were analyzed using paired samples *t*-tests.

### Clinical Evaluation

#### Participants

Following development and evaluation of the stress classifier, 16 participants [13 males; average age 39.8 ± 10.5 (SD)] were enrolled for participation in the clinical evaluation study of the classifier and associated mHealth application, which lasted 8–10 weeks for each individual. Participants were recruited from patients at the Philadelphia VA Medical Center who reported significant difficulties with anger and/or stress and indicated a willingness to participate in a research study. Exclusion criteria consisted of: currently active duty military; moderate or severe TBI; severe mental impairment as assessed in their electronic medical record; and/or functional limitations preventing use of a mobile device.

#### Experimental Procedure

Following written-informed consent, participants were randomly assigned to the experimental or control group and completed the DASS, PROMIS-Anger, and PTSD Checklist-Military (PCL-M) questionnaires. The control group (*n* = 6) received standard CBT; the experimental group (*n* = 10) received standard CBT integrated with the stress classifier and mobile application (see Physiological Measures). Following initial assessment, an appointment was made to begin treatment within 1–2 weeks. All treatment was administered in an individual format by the study clinicians, who are licensed mental health professionals. The study clinicians were directed to use standard CBT treatment manuals (32) as a foundation for CBT while using clinical judgment to determine what content to cover in each session and how many sessions to schedule. Typical treatment following this approach was expected to last 8–10 weeks. This approach was chosen rather than utilizing a fixed protocol in order to represent routine clinical practice.

Sessions involved a weekly, in-person meeting, which lasted 60 min. Patients were asked to keep a log of daily activities, summarizing key stress/anger events that occurred, and present this written report to therapists during their session. Weekly sessions continued until: (a) the participant and clinician jointly determined that there was significant clinical improvement; (b) it was judged by the therapist that no further improvement was likely to occur; or (c) the participant discontinued therapy. Compliance in the experimental group was quantified by use of the mobile application. One month following the completion of treatment, participants were asked to return for a follow-up visit to complete the DASS, PROMIS Anger, and PCL-M questionnaires.

#### Qualitative Measures

Participants completed the DASS and PROMIS anger scale at their initial assessment and following therapy completion; the DASS-Stress and PROMIS anger scales were considered primary outcome metrics. Participants in the clinical study also completed the PCL-M, which is a 17-item continuous severity measure that corresponds to the 17 DSM-IV criteria for PTSD (33). Respondents indicated the extent to which they had experienced each symptom described in the past month using a 5-point scale, from 1 (not at all) to 5 (very often). The PCL-M was considered a secondary outcome metric in the clinical study.

#### Physiological Measures

An mHealth application and stress classifier were used for data collection in the clinical study. The mHealth application was implemented in Android on a Samsung Galaxy S4 phone and received data from the E3 band (Empatica, Milan, Italy), classified stress using the algorithm developed in the classifier study, alerted the user when stress was detected, and presented stress mitigation techniques to the user, such as breathing exercises. The E3 band sent PPG, EDA, temperature, and accelerometer information to the mobile application *via* Bluetooth 4.0. A web-based provider portal that resided on a secure cloud server was also implemented and allowed the provider to view physiological data for individual patients and enter reminders (e.g., complete your cognitive restructuring homework) or focus points (e.g., practice breathing), which were sent to the mobile application.

#### Data Analysis and Statistics

Non-parametric statistical analysis was used to compare within groups measures across the two timepoints (initial and final assessment) and consisted of Wilcoxon signed-ranks tests with significance set to 0.05. Between groups differences were assessed using Mann–Whitney *U* tests with significance set to 0.05. All statistical testing was done in SPSS software version 18.

## Results

### Classifier Study

The sociodemographic factors in the initial classifier study are listed in Table 1. The average age of the participants was 25.7 ± 6.2 (SD) years, and participants had an average of 3.4 ± 2.0 (SD) years of post-secondary education.

**Table 1:** **List of sociodemographic factors in the classifier study sample**.

	Study sample% (*n*)
**Gender**	
Male	68.6 (24)
Female	31.4 (11)
**Age group**	
18–21	25.7 (9)
22–25	40.0 (14)
>25	34.3 (12)
**Education**	
High school diploma/GED	14.3 (5)
Some college/university	25.7 (9)
Bachelor’s degree	37.1 (13)
Graduate degree	22.9 (8)

**Figure 1:**
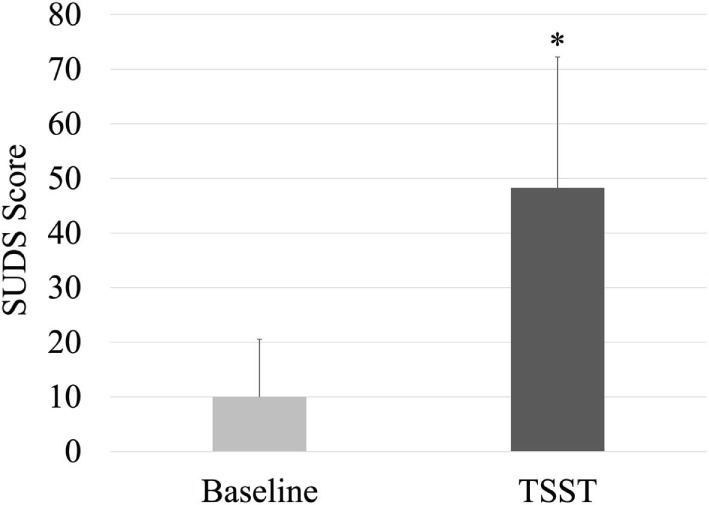
**Self-reported distress at baseline and following the TSST**. Mean + SD shown. *p < 0.001.

Subjective Units of Distress Scale scores are shown in Figure 1. As compared to baseline, the TSST elicited a significant increase in perceived stress (*p* < 0.001, *d* = 1.44). Baseline cortisol in the study subset was 0.39 ± 0.33 (SD) μg/dL, which did not differ following the TSST at a level of 0.44 ± 0.39 (SD) μg/dL (*p* = 0.29, *d* = 0.16) *via* paired samples *t*-test.

Depression, anxiety, and stress scale scores are shown in Table 2. Stress, depression, and anxiety scores were considered normal (25). The PROMIS anger score was 50.1 (7.2).

**Table 2:** **Mean (SD) DASS scores in the classifier-development group**.

DASS – stress	DASS – depression	DASS – anxiety
7.4 (6.2)	5.1 (6.1)	3.9 (4.6)

**Figure 2:**
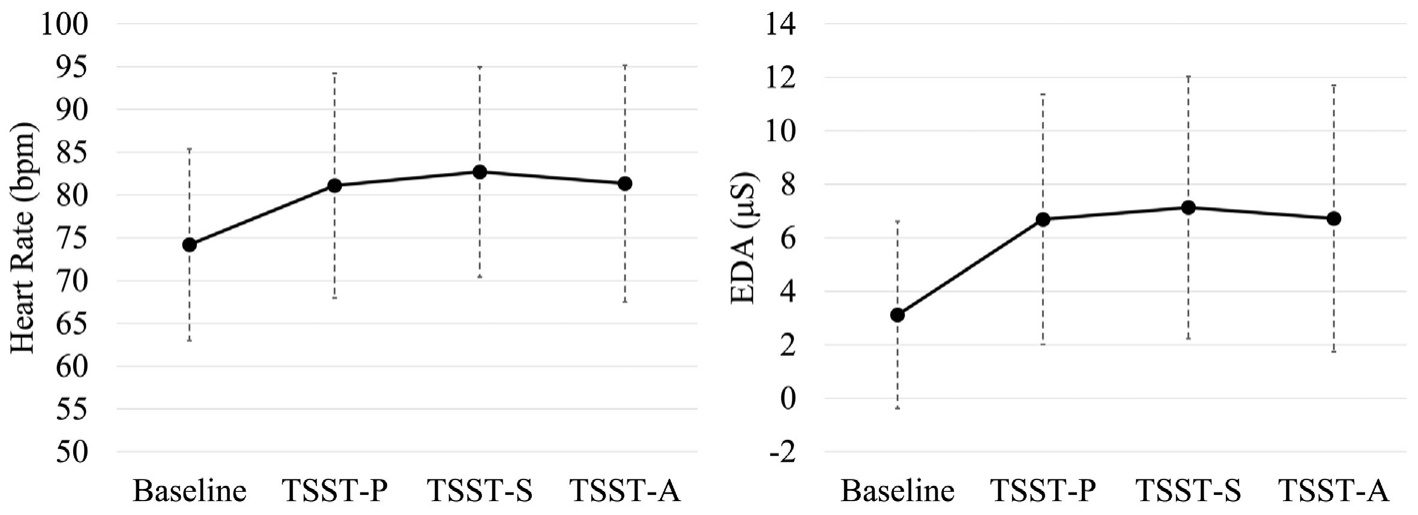
**Task-dependent heart rate and skin conductance measures across participants**. **(Left)** Includes heart-rate estimate distributions; TSST-S, TSST-A, have notably high HR distributions, whereas baseline tends to be low. **(Right)** includes electrodermal activity estimate distributions; the baseline conductance is relatively low, whereas TSST-S and TSST-A, are relatively high. Shown are group means ± SD.

Noise or data loss affected 4/35 participants’ physiological data, which were removed from analysis. Each task phase in the experiment was regarded as having distinct ground truth values for whether the participant would be considered stressed or not stressed. The TSST-S and TSST-A phases of the TSST were considered to be psychological stress phases. The baseline resting task was considered to be a non-psychological stress phase. For the TSST-P task no assumption of stress vs. non-stress was made. Figure 2 shows the distributions of (non-normalized) HR (left) and skin conductance (right) data vs. task for all participants.

**Figure 3:**
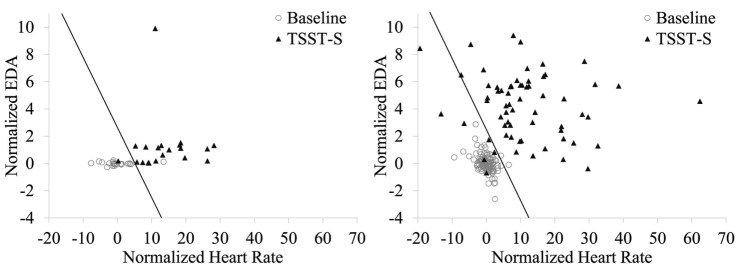
**Stress vs. non-stress classifier using baseline-normalized HR and EDA features during the baseline and TSST-S segments**. Stress classification using the E3-collected data is shown at left and with the Biopac-collected data shown at right. The decision boundary is shown as a line; data points to the left of this boundary were classified as non-stress.

Based on the distributions, baseline-normalized HR and EDA means were used for stress vs. non-stress classification. Figure 3 shows the classification results of training the stress vs. non-stress classifier using 75% of the E3 physiological data. For the E3 data, the training accuracy was 97.1%. Test set accuracy on the remaining 25% of the data were 91.7%. The hit rate on the test set was 100%, and the false-alarm rate was 12.5%. For the Biopac data, the testing accuracy was 95.1%, the hit rate was 89.1%, and the false alarm rate was 1.7%.

### Results from Clinical Assessment

The sociodemographic factors in the clinical evaluation are listed in Table 3. The average age of the participants was 39.7 ± 10.5 (SD) years and most were US Army veterans.

**Table 3:** **List of sociodemographic factors of study sample**.

	Study sample% (*n*)
**Gender**	
Male	81.2 (13)
Female	18.8 (3)
**Age group**	
20–29	6.2 (1)
30–39	50.0 (8)
40–49	25.0 (4)
50–59	18.8 (3)
**Education**	
High school diploma	25.0 (4)
Some college/university	56.2 (9)
University degree	18.8 (3)
**Military branch**	
Army	68.8 (11)
Navy	12.5 (2)
Air force	12.5 (2)
Marines	6.25 (1)

Nine individuals in the study dropped out prior to completion of therapy and follow-up visit. A Mann–Whitney test indicated that individuals in the experimental group completed a significantly greater number of therapy sessions (*p* = 0.016, *d* = 1.34) at an average of 7.2 ± 1.6 (SD) sessions as compared to 3.4 ± 2.4 (SD) in the control group. The remaining participants that completed the study included five in the experimental group and two in the control group. One participant in the experimental group that completed the study did not use the mHealth application but completed standard CBT and was reassigned to the control group.

Depression, anxiety, and stress scale scores are shown in Table 4. For the initial assessment, stress and depression for the participants was in the 96^th^ percentile, and anxiety was in the 99^th^ percentile as compared to a normative sample (34). Anxiety scores were considered extremely severe, while stress and depression scores were in the severe range (25). No differences between the control and experimental group were observed during the initial assessment for stress scores (*p* = 0.616, *d* = 0.21), depression (*p* = 0.964, *d* = 0.09), or anxiety (*p* = 0.682, *d* = 0.29) as assessed by Mann–Whitney testing.

**Table 4:** **Mean (SD) DASS assessment scores**.

	Initial assessment			Follow-up		
DASS scale	Stress	Anxiety	Depression	Stress	Anxiety	Depression
Control	29.7 (12.6)	28.3 (11.4)	27.3 (11.3)	30.7 (4.2)	22.7 (6.4)	16.7 (10.1)
Experimental	27.8 (6.7)	22.2 (12.4)	20.6 (5.9)	16.0 (5.6)^a^	11.0 (8.1)^a^	14.5 (6.2)

*^a^indicates significant difference between groups*.

The follow-up assessment was completed by four participants in the experimental group and three participants in the control group. A significant reduction in stress (*p* = 0.032, *d* = 1.61) and anxiety (*p* = 0.050, *d* = 1.26) was observed between the experimental and the control group but not for depression (*p* = 0.719, *d* = 0.29) per Mann–Whitney testing. Within groups, the control group showed no significant changes in DASS scores for stress (*p* = 0.593), anxiety (*p* = 0.109), or depression (*p* = 1.000) between the initial assessment and follow-up as assessed by Wilcoxon signed-ranks tests. The experimental group trended toward a significant decrease in stress (*p* = 0.068) and depression (*p* = 0.068) between timepoints but not anxiety (*p* = 0.144) as assessed by Wilcoxon signed-rank tests.

Patient-reported outcomes measurement information scale anger scores are shown in Table 5. No differences between the control and experimental group were observed at the initial assessment (*p* = 0.703, *d* = 0.20), but follow-up scores indicated a significant reduction in anger for the experimental group (*p* = 0.046, *d* = 1.41) as assessed by Mann–Whitney testing. Within groups, no difference in anger was observed for the control group (*p* = 0.715) or the experimental group (*p* = 0.109) as assessed by Wilcoxon signed-ranks testing.

**Table 5:** **Mean (SD) PROMIS Anger scores**.

	Initial assessment	Follow-up
Control	66.6 (7.1)	71.5 (9.7)
Experimental	66.1 (8.7)	55.4 (2.4)^a^

*^a^indicates significant difference between groups*.

PTSD checklist-military scores are shown in Table 6. No differences between the control and experimental group were observed at the initial assessment (*p* = 0.639, *d* = 0.16) or at follow-up (*p* = 0.480, *d* = 0.57) as assessed using Mann–Whitney testing. Within groups, no difference in PCL-M scores was observed for the control group (*p* = 0.285) or the experimental group (*p* = 0.144) as assessed by Wilcoxon signed-ranks testing.

**Table 6:** **Mean (SD) PCL-M Scores**.

	Initial assessment	Follow-up
Control	60.8 (14.1)	51.3 (5.5)
Experimental	59.7 (12.2)	43.5 (18.0)

## Discussion

The current series of studies shows the feasibility of creating an individualized, physiological classifier of stress with a high degree of accuracy compatible with different sensor suites. The use of such an algorithm in an mHealth application (35) may reduce symptoms of stress and anger in a small clinical population, increase the number of CBT sessions individuals will attend, and decrease their dropout rate. Given the large number of individuals that experience mental health disorders and the unmet need for treatment, especially in developing nations, such mobile approaches have the potential to provide or augment treatment in the absence of standard, in-person care (36). However, most commercially available apps targeting mental health remain untested (22, 37).

Classification of stress was based on features gathered from a large user group undergoing the TSST, which has one of the highest effect sizes for eliciting stress and associated cortisol responses in laboratory settings (38). Stress classification was based on cardiovascular and electrodermal inputs (3), which showed high variance due to individual differences (19), and was addressed by individual baseline normalization. The psychoendocrine reaction to life stressors, or stressors outside of the laboratory setting, such as bereavement, declining health, or flashbacks in PTSD, are likely of higher duration and intensity than laboratory stressors (39). Therefore, the algorithm and decision boundary developed using acute socio-evaluative stress in the current work may underperform for more severe stressors associated with MDD, PTSD, or other forms of mental health disorders. For instance, the DASS and PROMIS-anger scores from the classifier-development study sample indicated a relatively low burden of stress, depression, anxiety, and anger as opposed to a relatively high burden of mental health symptoms in the clinical evaluation study sample. In addition, veteran post-traumatic stress is often comorbid with depression, which has recently been shown to be associated with intensified anger (2). Anger has been acknowledged as the most prevalent veteran readjustment problem (14). Interestingly, the use of an mHealth application focused on stress identification and reduction in conjunction with CBT reduced metrics of anger and anxiety in addition to stress in a small clinical sample.

The overall dropout rate from CBT has been reported to be between 25–40% for depression (12, 13). In the current study, over 50% of the participants discontinued therapy, early. This higher dropout rate may be due to characteristics of the veteran population or the high burden of mental health symptoms in the study sample. For example, previous research has indicated that medication compliance among veterans is relatively low (40). The high dropout rate likely also reflects that many of the participants were experiencing periods of acute stress and were often preoccupied with these stressors. The availability of validated mHealth applications that individuals could use within the context of their daily lives would help to address this issue. Within the sample, those who used the mobile application and stress algorithm were more likely to complete the study and demonstrated reduced stress and anger as compared to the control group. This reduced stress may result from an increased awareness of their stressors due to the alerts provided through the mobile application to the user (41), or the use of the guided breathing exercises within the application (42).

Future research will include further accuracy refinement through reduction in environmental noise, and a method to learn individual user stress thresholds (19). Additional operational testing to reduce environmental noise is being conducted in order to determine the changes in classifier false alarms and misses when collecting data in different temperatures and while performing different physical activities (43), ranging from typing on a keyboard to walking or running. The 2-feature linear model trained with stochastic gradient descent employed in this effort has the advantage of including a bias term to tune the decision boundary threshold on the stress vs. non-stress classifier to allow adjusting the tradeoff between hit and false-alarm rates, ultimately generating an individualized threshold for each user.

The low sample size in the clinical evaluation and the high dropout rate represent a limitation in the current study. Even though there have been an estimated 180,000 cases of US military veterans with PTSD over the past two decades (44), many do not seek care (45). Additional challenges include long wait times experienced in the VA medical system (46), low participation rates in clinical studies (47), and a high dropout rate during CBT. Further data and objective outcome measures are needed to validate the observed reductions in stress, anger, and anxiety symptoms in the study sample.

The capability to classify individual physiological stress in a mobile environment has additional uses outside of veteran mental health treatment, including military or medical training (48). For example, training instructors could remotely and simultaneously monitor objective stress status for individual trainees during live training sessions and act on the information in real time (49). In addition, instructors could identify individual trainees that tend to have more intense stress responses than others during training scenarios to provide targeted coping and resilience training interventions (50). Beyond training, additional applications for this capability include stress research for laboratory and field settings, chronic disease monitoring for tracking outpatient health and long-term data capture to inform care, and objective, real-time user experience evaluations. mHealth applications and wearable physiological sensors have the potential to analyze and present meaningful data to better manage and optimize general health and specific health conditions. However, high quality, wearable devices and robust, validated algorithms remain a necessary component to realizing the potential of this technology.

## Author Contributions

BW analyzed data and wrote the manuscript. GC and BW developed the algorithm for stress. SD and DJ developed the mobile application and managed the experiments. PG designed the clinical evaluation. SK and JG conducted the clinical evaluation.

## Conflict of Interest Statement

GC, BW, SD, DJ (2014) System, method, and computer program for the real-time mobile evaluation of physiological stress. US Patent Pending #10878-023.
